# Condyloma of the neovaginal vault successfully treated with topical cidofovir therapy

**DOI:** 10.1016/j.jdcr.2023.09.016

**Published:** 2023-09-26

**Authors:** Jose L. Cortez, Danielle N. Turner, John R. Durkin, Mary E. Logue

**Affiliations:** a Department of Dermatology, University of New Mexico, Albuquerque, New Mexico; b University of New Mexico, School of Medicine, Albuquerque, New Mexico

**Keywords:** condyloma acuminata, LGBTQ care, neovagina, topical cidofovir, transgender care

## Introduction

There is no consensus for treating condyloma arising in the neovaginal vault in transgender women following gender-affirming vaginoplasty. Prior reports have documented successful treatment of neovaginal condyloma with carbon dioxide (CO2) laser, topical imiquimod, and topical trichloroacetic acid (TCA). Additionally, topical cidofovir has been cited in the literature as efficacious for the treatment of external genital warts in cisgender patients. Here, we discuss a case of condyloma of the neovaginal vault arising de novo in a transgender woman who was successfully treated with 1% topical cidofovir with sustained clearance.

## Case report

A 43-year-old transgender female with no prior history of sexually transmitted infections (STIs) presented with neovaginal lesions 18 months postpenile inversion vaginoplasty. She initially presented to her obstetrics and gynecology with worsening vulvar irritation and pain that prevented her from using her dilators and diagnosed with bacterial vaginosis. During her speculum exam, her obstetrics and gynecology biopsied lesions that were histologically consistent with condyloma acuminata; she was then referred to Dermatology. Her external genitalia was markedly inflamed and macerated with superficial erosions and secondary white to gray exudate. A speculum exam revealed several clusters of fleshy, inflamed verrucous papules and plaques within the posterior and lateral walls of the neovaginal vault. The patient’s performing vaginoplasty surgeon was consulted to discuss treatment options, given the complexities around the anatomy and microbiome of the neovagina. The decision was made to begin daily application of compounded topical 1% cidofovir cream (ChemistryRx). Of note, although TCA was an initial treatment of choice, it was unable to be obtained in a reasonable time due to supply chain issues. Destructive modalities such as cryotherapy or CO2 laser would carry a higher risk of damaging the neovagina and are difficult to perform on the internal vagina. Topical 5-fluorouracil and imiquimod could both exacerbate the inflammation and erosion that was already present. Additionally, imiquimod has a higher risk of systemic absorption on mucosal surfaces with unwanted side effects such as fevers, chills and flu-like symptoms. As cidofovir is virucidal and can be compounded to a lowest effective concentration to directly target the warts, it was felt this option would be the optimal approach to spare healthy tissue and minimize the risk of localized and/or systemic side effects.

**Fig 1:**
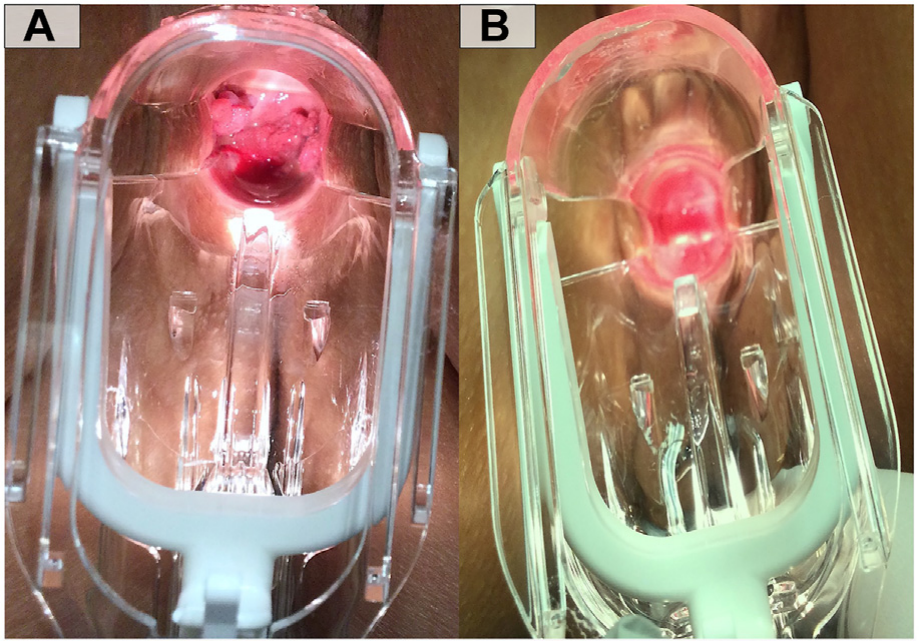
Clinical presentation of condyloma of the neovagina of a transgender woman. **A,** Pretreatment speculum examination demonstrates *pink* papillomatous papules in the neovaginal vault. **B,** Post-treatment speculum examination with complete resolution of *pink* papillomatous papules in the neovaginal vault.

After the first 2 weeks of once daily application of cidofovir 1% cream, patient reported a noticeable decrease in her condyloma burden during manual self-exams. She was unable to manually apply cream to the posterior vault, so we started using her smallest gauge dilator to successfully apply the cream to this area. After a total of 6 weeks of treatment, her follow-up speculum exam confirmed no residual condyloma clinically (Fig 1). The only side effect of her therapy was worsening of her initial vaginitis which was treated with pulse dosing of metronidazole 500 milligrams for 7 days followed by fluconazole 150 milligrams on day 8, every 2 weeks, as well as metronidazole 0.75% cream, miconazole 2% cream, and tacrolimus 0.1% ointment for twice daily application to the external genitalia during flares. As maintenance, zinc oxide 10% cream was used as a skin protectant and 99% water based wipes for hygiene. At this time, patient remains clinically clear of condyloma.

## Discussion

Transgender individuals experience disparities in care that can be detrimental to their overall physical and psychosocial health. Higher rates of STIs, violence, and mental health conditions have been reported in this population.^1^ Dermatologists can play an important role in the screening, prevention, and treatment of STIs in transgender individuals.^2^ In transgender women, diagnosis of condyloma can be challenging and requires an understanding of gender-affirming vaginoplasty and the sexual behaviors of these individuals.^3^ Additionally, there is a paucity of information on how to treat condyloma in transgender individuals which requires consideration of the delicate nature of the skin used to create the neovagina.

Prior studies have documented the new onset of condyloma in the transplanted neovaginal skin of transgender women. Both Yang et al and Liguori et al commented on the rarity of literature on condyloma in transgender women and a lack of consensus on treatment.^4^^,^^5^ Matsuki et al reported successful clearance of condyloma in the neovagina of a transgender woman with CO2 laser followed by topical imiquimod for residual warts; this was the first report to document the successful clearance of condyloma with imiquimod.^6^ Fein et al subsequently reported successful treatment of biopsy-proven neovaginal condyloma in a transgender woman with TCA.^7^ Individuals in both reports had sustained clearance.

The New England Journal of Medicine (NEJM) published a case series of 3 cisgender male and female patients with condyloma who were successfully treated with topical 1% cidofovir. All patients were noted to have significant improvement after a single week of therapy and remained free of recurrence at 6-12 month follow up.^8^ Subsequently, a systematic review by Jung et al demonstrated that efficacy of topical cidofovir as a therapeutic agent for the treatment of external genital warts was comparable to that of conventional therapies such as imiquimod with no differences in recurrence of warts or in severe adverse events.^9^ Therefore, these studies suggested topical cidofovir could serve as a treatment option for condyloma arising in the neovaginal vault.

Herein, we described a case of condyloma in a transgender woman with no history of condyloma prior to vaginoplasty. To the best of our knowledge, this individual represents the first case of successful treatment with topical cidofovir of condyloma presenting de novo in the neovagina of a transgender woman. This case again underscores the important role dermatologists play in screening, prevention, and treatment of STIs in transgender individuals.^2^ Transgender women who present with new onset condyloma should also be screened for other STIs. Treatment of neovaginal condyloma should consider the anatomy and delicate skin of the neovagina. Our case also highlights the unique nature of the neovaginal microbiome and the importance of successfully managing vaginitis to optimize dilation exercises that are vital in maintaining the neovagina’s postoperative patency. In summary, topical 1% cidofovir cream can be considered as a viable, alternative treatment to CO2 laser, imiquimod, and TCA.

## Conflicts of interest

None disclosed.
